# Structural changes induced by ultrasound improve the ability of the myofibrillar protein to bind flavor compounds from spices

**DOI:** 10.1016/j.ultsonch.2023.106510

**Published:** 2023-07-03

**Authors:** Xiangxiang Sun, Yumei Yu, Ahmed S.M. Saleh, Xinyu Yang, Jiale Ma, Ziwu Gao, Wenhao Li, Zhenyu Wang, Dequan Zhang

**Affiliations:** a Key Laboratory of Agro-Products Processing, Institute of Food Science and Technology, Chinese Academy of Agricultural Sciences, Ministry of Agriculture and Rural Affairs, Beijing 100193, China; b College of Food Science and Engineering, Northwest A&F University, Yangling 712100, China; c Department of Food Science and Technology, Faculty of Agriculture, Assiut University, Assiut 71526, Egypt

**Keywords:** Myofibrillar proteins, Chicken meat, Flavors, Ultrasonic, Spices

## Abstract

Effects of ultrasound (UT) treatments on the structural, physicochemical, and functional properties of myofibrillar proteins (MPs), as well as their ability to bind to flavor compounds from spices, were investigated. The results demonstrated that UT treatment enhanced surface hydrophobicity, SH content, and absolute ζ-potential value of the MPs. Atomic force microscopy analysis displayed formation of MPs aggregates with small particle size in the UT-treated MPs samples. Meanwhile, UT treatment could improve the emulsifying properties and physical stability of MPs’ emulsion. Additionally, the MPs gel network structure and stability significantly improved following UT treatment. Changes in the structural, physicochemical, and functional properties enhanced the ability of MPs to bind to flavor substances from spices depending on the duration of UT treatment. Furthermore, correlation analysis showed that the ability of myristicin, anethole, and estragole to bind to MPs was highly correlated with surface hydrophobicity, ζ-potential value, and α-helix content of MPs. The results of this study may help in understanding the relationship between the changes in MPs properties during the processing of meat products and their ability to bind to flavors from spices, thereby improving flavors retention and taste of processed meat products.

## Highlights

• Myofibrillar proteins (MPs) aggregates existed in smaller form after ultrasound (UT).
• Changes in characteristics of MPs relied on UT treatment duration.
• The ability of MPs to bind to spices flavors enhanced after UT treatment.
• The UT demonstrated potential for producing flavors-rich meat products.

## Graphical abstract

**Figure:**
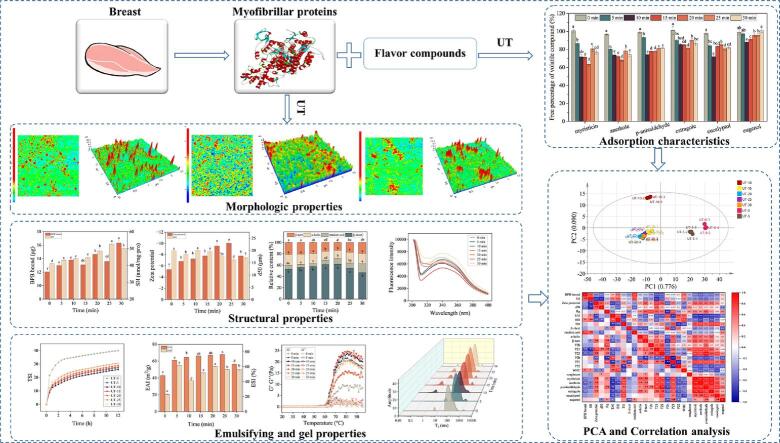

## 1 Introduction

Myofibrillar proteins (MPs) are the most important protein in meat and meat products, which account for more than 50% of the total protein content [1]. Additionally, the MPs are related to textural, sensory attributes, and flavor characteristics of meat and meat products [2]. Conditions such as temperature, oxidation, freezing, and pH could alter the microstructure of MPs [3]. Also, processing methods and conditions affect the protein structure [4]. The changes in protein structure influence the protein binding to volatile compounds. For example, the incubation at 25 and 37 °C could enhance the binding ability of myosin to aldehydes, while 60 °C promoted the release of aldehydes from myosin [5]. Additionally, the hydrophobic bonding sites, hydrogen-bonding sites, and electrostatic effects were enhanced by the ultrasonic (UT) treatment [1]. Meanwhile, the ability of the protein to bind with flavor compounds varies depending on the characteristic of MPs as well as the nature of flavor compounds [2]. The capacity of the protein to bind to volatile compounds from spices (VCS) has received less attention in earlier research [6], [7] than its ability to bind to aldehydes, ketones, ethyl esters, and other chemical volatile substances. Volatile substances, such as eugenol, p-anisaldehyde, eucalyptol, anethole, myristicin, and estragole, could cover up offensive odors and give meat products a clove, cooling, anise, sweet, herbal scent. Therefore, understanding the ability of the protein to bind to VCS under various processing conditions is important for predicting and controlling meat flavor.

UT technology, a type of non-isothermal technology, has been employed extensively in the meat industry during the past ten years and is known to be a safe and effective food processing technique [1], [8]. The UT could improve the digestibility of pork MPs, create a greater number of smaller peptides, enhance the particle size distribution to become more uniform, reduce the aggregation of MPs, and improve the affinity between furan compounds and MPs [1], [8], [9]. However, negative changes can due to inappropriate UT conditions. For example, UT treatment at a power higher than 200 W enhanced protein aggregation and reduced enzyme activity [10]. In another study, Wang et al. [11] found that UT treatment for 6 and 10 min was not enough to make a significant change in MPs solubility. Therefore, to positively alter the structural and functional characteristics of MPs and increase their capacity to bind to volatile compounds, the UT treatment conditions should be optimized. Additionally, the evaporation of flavor components from meat products such as braised meat, roast meat, and dry cured meat can be prevented by improving MPs' ability to bind to volatile compounds. Previous studies have reported the influence of UT treatment on protein properties. However, to the best of our knowledge, studies performed to investigate the effects of UT treatment on structure, rheological, emulsifying, and gel properties of MPs, as well as the ability of the MPs to bind to VCS are limited. Therefore, the aim of this study was to optimize UT treatment conditions to modify structural and functional properties of MPs to improve their ability to bind to flavor compounds from spices.

## 2 Materials and methods

### 2.1 Materials and reagents

Fresh chicken breast meats were offered by Yixingzhang Daokou braised chicken Co., LTD, Huaxian, Henan, China. The spices flavor compounds’ standards, including eucalyptol, anethole, p-anisaldehyde, eugenol, myristicin, and estragole, were purchased from Sigma-Aldrich Co., LTD (MO, USA). Buffer A (pH 6.8) included KCl (0.1 M), MgCl_2_ (2 mM), ethylene glycol tetraacetic acid (EGTA, 2 mM), and K_2_HPO_4_ (20 mM). Buffer B (pH 6.8) consisted of KCl (0.1 M), MgCl_2_ (2 mM), EGTA (2 mM), K_2_HPO_4_ (20 mM), and 10% Triton X-100. Buffer C (pH 6.25) included NaCl (0.6 M) and Na_2_HPO_4_ (50 mM). Buffer D consisted of ethylenediaminetetraacetic acid (EDTA, 10 mM) and KH_2_PO_4_ (0.1 M). All chemicals and reagents used in the study were of analytical grade.

### 2.2 Myofibrillar proteins (MPs) extraction

Myofibrillar proteins were extracted as described by Sun et al. [2]. Briefly, the minced sample and buffer A (1/10, w/v,) were mixed, homogenized for 30 s (twice), and centrifuged for 15 min (12096 × g, 4 °C, CR21N, Hitachi High-Technologies Corporation, Tokyo, Japan). The sediment was washed twice with buffer B and KCl (0.1 M), respectively. Pierce™ BCA Protein Assay Kit, offered by Thermo Fisher Scientific Inc. (Waltham, USA), was used to analyze the protein concentration of MPs.

### 2.3 UT treatment

The MPs solution was diluted to a protein concentration of 10 mg/mL by buffer C. Ten mL of MPs solution (10 mg/mL) was subjected to UT treatment at a power of 500 W for 0, 5, 10, 15, 20, 25, and 30 min; respectively, in an UT equipment (40 KHz, KQ-500E, Kunshan ultrasonic instrument co., Ltd, China). The obtained samples were marked as UT-0, UT-5, UT-10, UT-15, UT-20, UT-25, and UT-30, respectively.

### 2.4 Surface hydrophobicity

Surface hydrophobicity of samples was measured as described by Chelh et al. [12]. Briefly, MPs solution was adjusted to a protein concentration of 1 mg/mL using buffer C. The MPs solution and bromophenol blue (BPB, 100 μL) were mixed, shaken (4 °C, 10 min), then centrifuged at 4000 × g and 4 °C for 15 min (CR21N, Hitachi High-Technologies Corporation, Tokyo, Japan). Absorbance of samples was determined using a microplate reader (Spark, Tecan Ltd, Switzerland) at 595 nm.

### 2.5 Reactive sulfhydryl (SH) content

The reactive sulfhydryl content was determined as described by Hamada et al. [13] with slight modification, using a microplate reader (Spark, Tecan Ltd, Switzerland). Briefly, MPs solution was adjusted to a protein concentration of 1 mg/mL using buffer C. Then, MPs solution (0.5 mL), buffer D (4.5 mL), and Ellman’s reagent (100 μL) were mixed, kept at 4 °C for 1 h, then the absorption at 412 nm was measured.

### 2.6 ζ-potential

Zeta potential of samples was measured as described by Sun et al. [2]. Briefly, MPs solution was adjusted to a protein concentration of 1 mg/mL using buffer C. Then, sample (1 mL) was loaded into a quartz colorimetric dish to analyze Zeta potential using a Nano ZS90 zetasizer (Malvern Instruments Ltd., UK).

### 2.7 Particle size distribution analysis

The MPs solution was adjusted to a protein concentration of 1 mg/mL using buffer C. Then, sample (1 mL) was dropped into a sample cell to analyze the particle size distribution using Microtrac S3500 particle size analyzer (Microtrac Ltd., USA).

### 2.8 Secondary structure analysis

Secondary structure analysis of samples was performed as described by Luo et al. [14] with slight modification. Briefly, freeze-dried powder sample and KBr (2:100) were mixed, compressed, then scanned in the range of 4000–400 cm^−1^ at a speed of 4 cm^−1^ using a Vertex 70 Fourier infrared spectrometer (Bruker Co., Ltd., Ettlingen, Germany). Peakfit 4.11 (Systat Software Inc., San Jose, CA) was used to analyze the data of 1600–1700 cm^−1^ (amide I band).

### 2.9 Fluorescence spectroscopy analysis

Fluorescence spectroscopy analysis was performed as described by Liu et al. [6] with slight modification, using F-380 (Tianjin Gangdong Sci.&Tech. Co., Ltd., Tianjin, China). The MPs solution was adjusted to a protein concentration of 1 mg/mL using buffer C. The slit widths for both the excitation and emission were 5 nm. The emission spectra were captured between 300 and 400 nm, with the excitation wavelength set at 285 nm.

### 2.10 Small-angle X-ray scattering (SAXS)

The MPs samples were analyzed using Anton Paar SAXS (Anton Paar Ltd., Austria) equipped with Vantec 2000 area detector. The MPs solution was kept in sealed cells before X-ray exposure. The incident monochromatic light wavelength λ, sample-to-detector distance, and X-ray source were 0.1542 nm, 541 mm, and CuKa radiation, respectively. Guinier analysis was used to get the values of the radius of gyration (Rg) from the scattering data [15].

### 2.11 Atomic force microscopy (AFM) analysis

The FAM analysis of samples was performed as described by Sun et al. [2]. The MPs solution was adjusted to a protein concentration of 0.25 mg/mL using deionized water and dropped onto the mica sheet, then observed using an atomic force microscope (NX10, Park Systems Ltd., Korea).

### 2.12 Emulsion properties analysis

Emulsion properties of samples was measured as described by Gao et al. [16]. Briefly, 10 mg/mL of MPs suspension and soybean oil (4/1, w/v) were mixed and homogenized for 30 s (twice). Then, 50 μL emulsion and 5 mL sodium dodecyl sulfate (SDS, 1 mg/mL) were mixed. The absorbance was measured (0 min, A_0_) and after 10 min (A_10_) using a microplate reader at 500 nm (SPARK, Tecan Ltd, Switzerland). Emulsifying activity index (EAI) and Emulsion stability index (ESI) were calculated as follows:

(1) $$EAI\left(m^{2}/g\right)=\frac{2\times 2.303\times A_{0}\times n}{C\times (1-\rho )\times 10000}$$

(2) $$ESI\left(\%\right)=\frac{A_{10}}{A_{0}}\times 100$$

where n is the dilution factor, C is the concentration of protein (g/mL), and *ρ* is the oil volume fraction (v/v) of the emulsion.

### 2.13 Turbiscan stability index (TSI) measurement

Turbiscan Lab Expert analyzer (Formulaction Inc., Toulouse, France) was used to analyze the physical stability of MPs emulsion. The sample (20 mL) was put into the test tube and scanned for 12 h at the speed of 0.5 h at 25 °C.

### 2.14 Rheological analysis

Rheological analysis of samples was performed as described by Sun et al. and Wang et al. [2], [17] with slight modification, using an MCR 301 (Anton Paar GmbH, Graz, Austria). Briefly, MPs solution was adjusted to a protein concentration of 5 mg/mL using buffer C. The MP solution was placed on the parallel plate with a gap of 100 μm.

#### 2.14.1 Flow properties

The apparent viscosity was analyzed in the shear rates range of 0.1–100 s^−1^ at 25 °C with a gap of 100 μm.

#### 2.14.2 Frequency sweep tests

The storage modulus (G′) and loss modulus (G′′) was analyzed in the angular frequency (ω) range of 0.1–10 rad/s at the strain amplitude of 0.5%.

#### 2.14.3 Temperature sweep tests

Samples were kept at 20 °C for 5 min, heated from 20 to 95 °C (5 °C/ min) at frequency of 0.1 Hz and strain amplitude of 0.5%.

### 2.15 Gel properties analysis

The MPs solution was diluted to a protein concentration of 40 mg/mL using buffer C, and heated from 25 to 80 °C at 1 °C/min, kept at 80 °C for 20 min, cooled in ice for 30 min, then stored at 4 °C.

#### 2.15.1 Low-field nuclear magnetic resonance (LF-NMR) analysis

The NMI20-Analyst low-field nuclear magnetic resonance analyzer (Niumag Electric Corporation, Shanghai, China) was used to analyze the relaxation time (T_2_) of samples. The instrument's parameters were as follows: SF (20 MHz), TW (3000 ms), TE (1 ms), NS (8), and NECH (5000).

#### 2.15.2 Water holding capacity (WHC)

Water holding capacity of samples was measured as described by Xia et al. [18]. The samples were centrifuged at 4 °C (10000 × g, 10 min) and the WHC was calculated as follows:

(3) $$WHC\left(\%\right)=\frac{W_{1}}{W_{0}}$$

where W_0_ and W_1_ were the weight before centrifugation and the final weight of the gel, respectively.

#### 2.15.3 Scanning electron microscopy (SEM)

SU 8010 (Hitachi, Japan) was used to analyze the morphological structure of samples at 50 keV as described by Wang et al. [17] at magnification of 12000 ×.

### 2.16 Adsorption properties analysis

Adsorption properties analysis of samples was performed as described by Sun et al. [2]. Briefly, each standard compound (1 g/L) and 8 mL buffer C or MPs solution were mixed to a concentration of 1 mg/L, then treated by UT for different durations. GC–MS (QP2010plus, Shimadzu Co., Ltd., Shanghai, China) was used to study the free percentages of VCS in the headspace in presence of MPs.

### 2.17 Statistical analysis

The statistical analysis of data was performed using the ANOVA (SPSS 21.0, SPSS Inc., Chicago, USA). The difference between means was analyzed by Duncan test (*P* < 0.05). Data were expressed as mean ± standard deviation (n = 3). Principal component analysis (PCA) and correlation analysis were run using Origin 2023 software (OriginLab Corporation, Massachusetts, USA).

## 3 Results and discussion

### 3.1 Surface hydrophobicity analysis

**Fig. 1:**
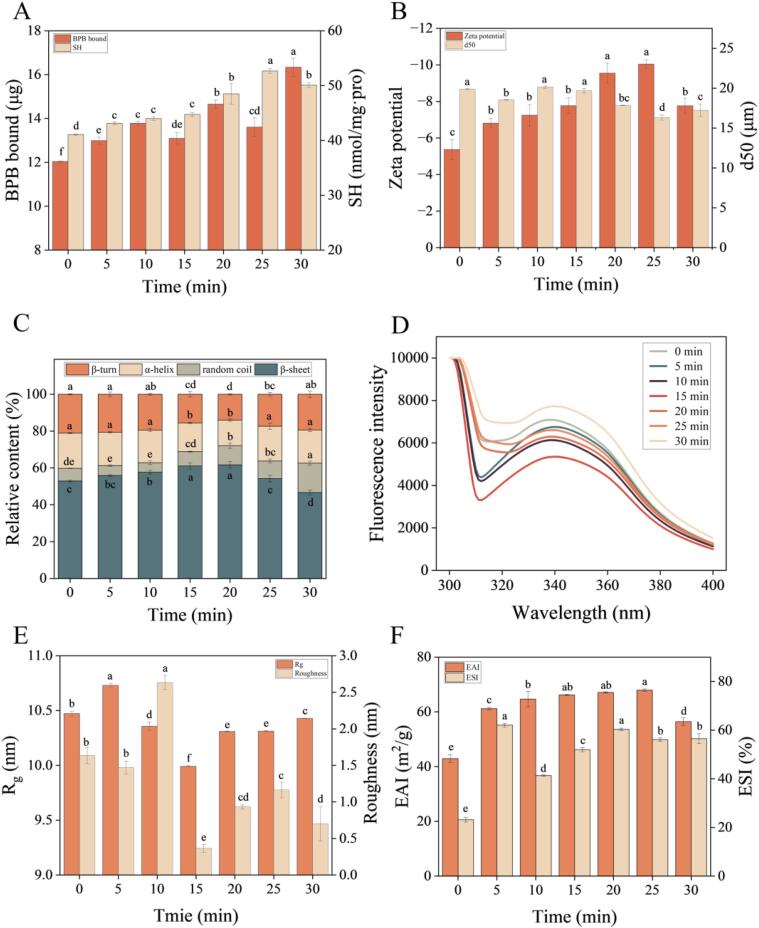
BPB bound and SH content (A), ζ-potential and particle size distribution (B), secondary structure (C), fluorescence spectra (D), Rg and roughness (E), and emulsifying properties (F) of MPs treated by UT. d50, the size of the particle for which 50% of the sample; Rg, radius of gyration; EAI, emulsifying activity index; ESI, emulsion stability index; columns with different letters indicate a significant difference (*P* < 0.05).

Surface hydrophobicity of samples is shown in Fig. 1A. Surface hydrophobicity could be used to characterize the conformational changes in proteins. Surface hydrophobicity of MPs was enhanced after UT modification, indicating that hydrophobic groups inside protein molecules were exposed, and the MP interaction was improved, improving the MP gel formation [19]. Similar results were found for white MPs of croaker surimi UT [1]. However, Li et al. [10] found that UT at power of 200–500 W reduced the surface hydrophobicity of sea cucumber gonad protein. Therefore, the changes in surface hydrophobicity may vary depending on UT treatment time, power, and protein source.

The large primary protein aggregates were disrupted and re-arranged, leading to buried hydrophobic groups being relocated to the surface, and a new surface was formed [20]. Notably, surface hydrophobicity of MPs reached a maximum value after UT treatment for 30 min, demonstrating that the long-time UT treatment improved the self-association of MPs. It was reported that the cavitation effect of UT causes changes in the hydrophobicity of protein surfaces [21]. Meanwhile, hydrophobic groups such as phenylalanine and tryptophan were highly exposed on the surface, resulting in strong hydrophobic interactions between the MP molecules [22].

### 3.2 The SH content

The SH content is an important indicator of changes in disulfide bonds [1]. The SH content of samples is shown in Fig. 1A. Compared with control group, UT-treated MPs had greater SH content, indicating that UT could increase the SH content. It may be due to that cavitation generated by UT promoted breaking of the disulfide bond or release of the thiol group embedded in the molecules [23]. Similar results were found after treatment of tuna MPs with UT at 100 W and 300 W [24] and MPs of chicken within 0–6 min of UT treatment [25]. Protein denaturing and unfolding after UT caused more SH exposure on the surface of the protein [2]. However, Liu et al. [9] found that UT at up to 400 W for 12 min could not change the MPs active SH groups.

When the UT prolonged from 0 to 5 min, the SH content of MPs increased, which can be explained by that the cavitation of UT led to the exposure of sulfhydryl groups [26]. In addition, no significant differences in the SH content of MPs was found within 5–15 min of UT treatment. This indicates that an appropriate UT treatment could maintain the unfolding of MPs molecules and the number of SH. Notably, the greatest SH content in MPs was found after 25 min of UT treatment, demonstrating that more interior SH groups were brought to the surface after UT treatment for 25 min. The input of long-time energy (UT treatment with more than 25 min) may cause aggregation of groups on the surface of molecules, resulting in a decrease of the SH content [9].

### 3.3 ζ-potential and particle size distribution

High absolute ζ-potential values indicate aggregation of protein and improvement of protein dispersion [27]. The ζ-potential of samples is shown in Fig. 1B. The UT treatment increased the absolute ζ-potential value, indicating that cavitation caused by UT changed the natural structure of MP molecules, resulting in more polar residues exposure on the surface of the MPs [28]. Interestingly, the highest absolute ζ-potential was found after 20 and 25 min of treatment, demonstrating storngly electrostatic repulsion stabilized MPs particles [29]. Similar results were found by Zhao et al. [1], who found more exposure of the negatively charged groups on the surface of MPs after UT treatment at power of 200–600 W, which caused an increase in the absolute ζ-potential value. However, in another study [11], it was found that UT decreased the absolute ζ-potential. The difference in the change of ζ-potential may be due to different ‘stress response’ and unfolding structure of MPs after UT treatment [30].

The particle size distribution of samples is shown in Fig. 1B. The changes in the MPs’ particle size distribution varied relying on the UT treatment duration. Compared with control group, UT-treated MPs for 25 min had lower particle size. This phenomenon may be due to that the cavitation bubbles caused by short-time UT treatment destroyed the agglomerates of MPs, resulting in a decrease in MPs’ particle sizes [30]. In addition, no significant differences in the particle size of MPs were found within 5–15 min of UT treatment. It was found that UT treatment improved the cross-linking and aggregation of MPs monomers or oligomers, leading to the formation of particles with larger size [31]. It is worth noting that the input of long-time energy (UT treatment for more than 15 min) caused a reduction of the MPs particle size. This could be due to that the turbulent actions, caused by long-time UT, changed the protein structure and depolymerized the agglomerates and masses into smaller particles, resulting in a reduction of the MPs particle size [27].

### 3.4 Changes in the secondary structure

The changes in secondary structure of samples are shown in Fig. 1C. The alteration of secondary structure varied depending on the UT treatment duration. As the UT duration prolonged, the β-sheet content first increased and then decreased. It may be due to that the cavitation produced by UT resulted in the hydrophobic regions’ exposure, causing reduction and fracture of intramolecular hydrogen bonds, leading to an increase in β-sheet content [32]. At the same time, the increase in β-sheet content indicated that protein–protein interactions were enhanced by appropriate UT, leading to the formation of intermolecular β-sheet structures [26]. Notably, the UT treatment for 15–20 min caused a remarkable increase in the content of β-sheet, and a significant decrease in the content α-helix of MPs (*P* < 0.05). This may be attributed to the mechanical vibration, caused by the bursting of cavitation bubbles, which produced a strong microflow and further resulted in a destruction in the protein secondary structure [33]. In addition, as UT treatment prolonged, protein molecules folded, and partial hydrogen bonds were enhanced, leading to the formation of ordered structure of α-helices and disruption of β-sheets [26].

### 3.5 Fluorescence spectroscopy analysis

Fluorescence spectroscopy could be used to understand the alterations in proteins’ tertiary structure. The fluorescence spectroscopy of samples is shown in Fig. 1D. The fluorescence intensity first decreased and then increased as UT treament prolonged, indicating that UT treatment could change the position of tryptophan residues, which were mostly enclosed inside the core (a hydrophobic environment) of MPs [26]. The decrease in fluorescence intensity may be explained by that the cavitation produced by UT caused an oxidation of tryptophan or protein unfolding, which embedded tryptophan inside MPs [1], [34]. In addition, the UT treatment for more than 15 min, the fluorescence intensity significantly enhanced, which may be due to modification of tryptophan residues in MPs, leading to a lesser exposure of tryptophan residues to the aqueous medium [35].

### 3.6 SAXS analysis

**Fig. 2:**
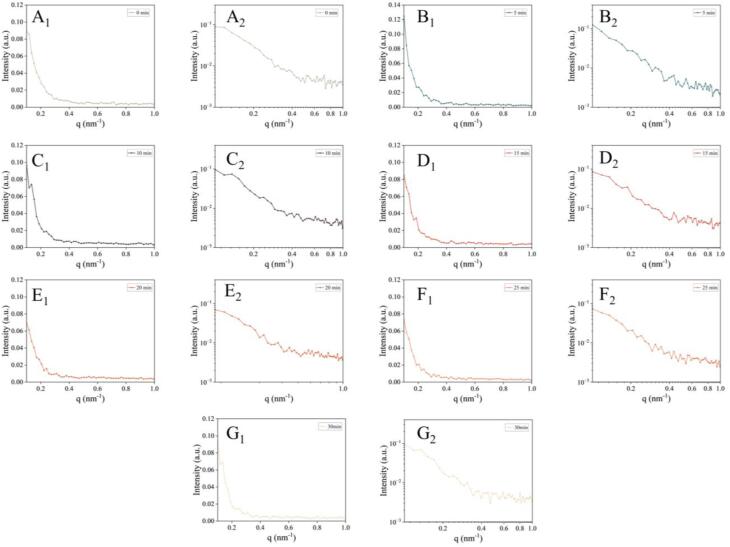
I-q patterns (1) and log–log plots (2) of double-logarithmic SAXS patterns of 709 UT-treated MPs. A, UT-0; B, UT-5; C, UT-10; D, UT-15; E, UT-20; F, UT-25; G, UT-30.

The SAXS is employed to the analyze structure of both ordered and disordered proteins [15]. It can be seen from Fig. 2(A_2_-G_2_) that all samples show good linearity on the SAXS patterns (log–log). Besides, some differences can be seen in the maximum value of the scattering intensity (Imax) of MPs after UT. Imax of MPs was enhanced after UT treatment for 5 min. Then, Imax first decreased and then increased the UT treatment prolonged from 5 to 30 min. This indicates that MPs underwent significant conformational changes after UT [15].

The radius of gyration (Rg), as the average electron-density-weighted, root-mean-squared distance of the scatters from the center of density in the molecule, could be used to analyze the changes in the overall size of MPs after UT [36]. The Rg value is smaller and protein is more compact [15], even though they have the same number of amino acid residues [36]. The Rg of MPs varied depending on the UT treatment duration (Fig. 1E). Appropriate UT treatment duration could enhance the Rg value of MPs, which may be attributed to unfolding resulted from the collapse of the secondary and tertiary structures. Due to their greatly stretched conformations, unfolded proteins have better properties for estimating average size than proteins with tightly packed core domains [37]. The UT-treated MPs for 15 min had a lower Rg value than the control group. This indicates that MPs had a more compact structure after UT treatment for 15 min. This phenomenon may be due to that the well-packed core (sub) domain of MPs became tighter after treatment, resulting in a lower Rg value [15].

### 3.7 AFM analysis

**Fig.3:**
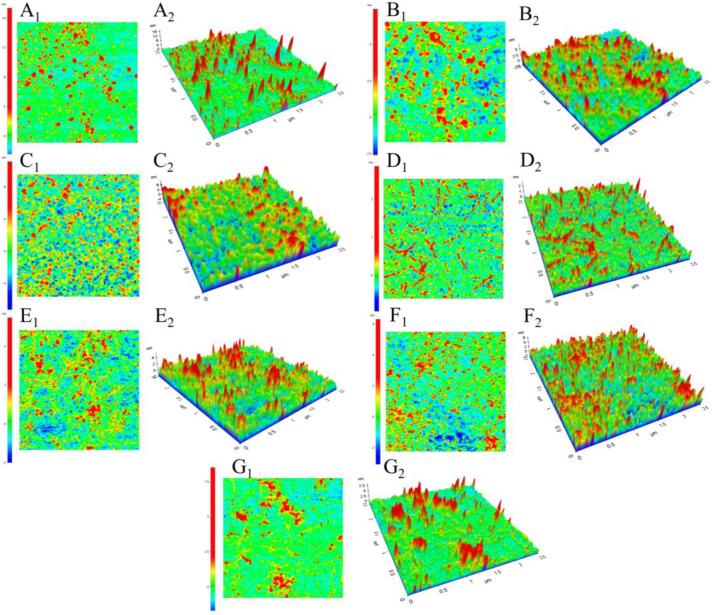
Two-dimensional morphology (1) and three-dimensional morphology (2) of MPs treated by UT. A, UT-0; B, UT-5; C, UT-10; D, UT-15; E, UT-20; F, UT-25; G, UT-30.

The atomic force microscope could be used to visualize the changes in microstructure of MPs after UT treatment for different durations, and the images are shown in Fig. 1E and Fig. 3. The MPs aggregates existed in smaller form after UT treatment. Due to cavitation caused by UT, which may break up the electrostatic interaction between charged clusters in MPs' amino acid residues, the height distribution was reduced. This, in turn, caused aggregates to depolymerize into oligomers and even monomers [38]. The system's inhomogeneity was reduced by acoustic cavitation produced by UT [29]. Compared with the control group, UT treatment for 15–30 min reduced the roughness of MPs, indicating that the particles of MPs were more evenly distributed (Fig. 1E). This could be as a result of the prolonged cavitation of UT, which could generate powerful physical forces such as shock waves, turbulence, and shear force that could efficiently break protein particles and reduce their particle roughness [39].

### 3.8 Emulsion properties

Emulsifying properties refer to the protein’s ability to disperse the oil phase into the water phase [34]. It can be seen from Fig. 1F that EAI of MPs was improved after UT treatment. The cavitation of UT improved the surface-to-volume ratio by altering MPs’ structure, making more proteins participate in the formation of interface layer, and improving the emulsification efficiency [24]. Similar results were found by Deng et al. [26] who reported that cavitation during UT enhanced the EAI, resulting in more integration of bubbles in the emulsified oil phase. It was also reported that EAI is affected by hydrophobic group exposure and protein molecular aggregation. However, UT treatment for more than 25 min caused a remarkable decrease in the EAI. It might be caused by a moderate degree of protein aggregation that has decreased conformational flexibility [33].

The ESI indicates the ability of proteins to remain at the water–oil interface after storage or heating of emulsion. Significant differences were found in the ESI of the untreated and UT-treated MPs. After UT treatment for 5 and 20 min, MPs had higher ESI than other treatments, indicating that appropriate UT duaration could enhance the ESI of MPs molecules, which is of great importance to further application of emulsions. This behavior might be caused by some internal hydrophilic group exposure and partial unfolding of protein structures, which boosted protein adsorption on the surface of oil droplets and increased ESI [40].

### 3.9 TSI analysis

**Fig.4:**
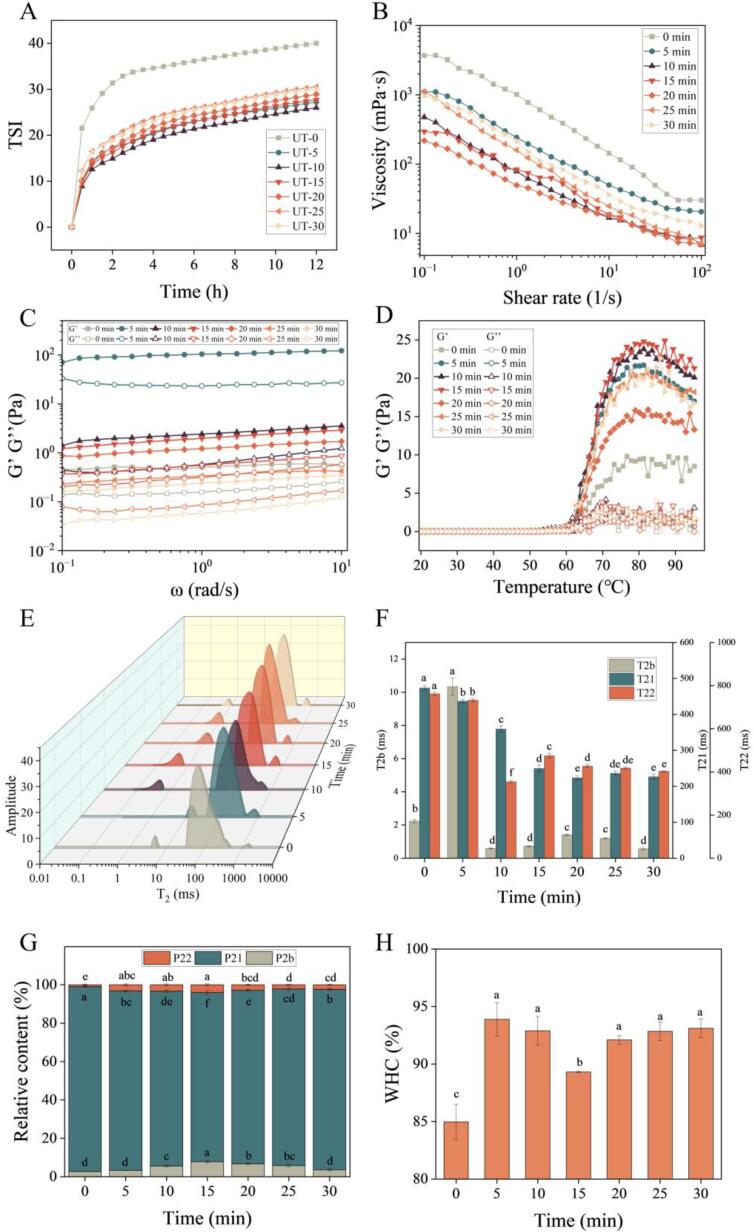
Turbiscan stability index (A), rheological properties (B-D), and gel properties (E-H) of MPs treated by UT; G’, storage modulus; G’’, loss modulus; columns with different letters indicate a significant difference (*P* < 0.05).

The TSI is an indicator for the physical stability of MPs emulsions during storage. The TSI of all samples increased as storage prolonged (Fig. 4A). The greater the change in TSI during the storage of emulsion, the worse the stability. Compared with the control group, UT-treated MPs emulsion had lower TSI, indicating that UT could improve the dispersion stability of MPs emulsion. This is consistent with findings of Kim et al. [41]. It is possible that a rise in TSI, which indicated more physically stable protein-based emulsions, was caused by cavitation of the UT, which produced uniformly tiny particles and produced the emulsion's stability [42]. Additionally, at the oil–water interfaces, the amphiphilic proteins create a stable amphipathic barrier by incorporating the hydrophobic groups into the oil phase and extending the hydrophilic groups into the watery phase, which resulted in high physically stable protein-based emulsions [16].

### 3.10 Rheological properties analysis

#### 3.10.1 Steady state analysis

Viscosity versus shear rate was analyzed to examine the protein–protein interactions in meat and the processing of meat [43]. Fig. 4B displays the results of a viscosity measurement to examine the fluidity of MPs following UT treatment. Viscosity of all samples decreased as the shear rate increased, demonstrating a shear-thinning feature. It might be because the molecular chain connections between MPs were broken down with increased shear rate, weakening frictional resistance and causing a reduction in viscosity [44]. In addition, UT could not change the shear-thinning property of MPs. Compared with the control group, UT-treated samples had lower viscosity, demonstrating that fluidity of MPs was enhanced by the UT treatment. This phenomenon might be caused by cavitation, in which the severe shearing forces generated by the UT caused the aggregates to depolymerize into small particles, damaging the protein chains and blocking protein interaction and aggregation [41]. It's interesting to note that UT treatment for 20 min caused the viscosity of the MPs to decrease faster than that of other UT treatment groups. Less head aggregation and tail cross-linking of MPs occurred after UT for 20 min, leading to a lower degree of MPs molecular chains interaction, and accordingly less resistance to flow seemed to exist in the 20 min UT-treated samples [2], [43].

#### 3.10.2 Dynamic rheology analysis

The information of network structure for MPs could be provided by storage modulus (G′) and loss modulus (G′′) versus frequency. It can be seen from Fig. 4C, G′ was much higher than G′′, indicating that the solid-like character and elasticity was the primary response in the gel system [40]. In addition, the G′ of MP gel was improved after UT, which reached the highest at 5 min of UT modification. In another study, Li et al. [42] found that 5 min of UT treatment could significantly improve G′ by 20%, and UT-treated MPs reached the highest G′ at 15 min. The results indicate that the structural strength of the gel network was enhanced after UT treatment. It might be because the UT cavitation bubble action promoted the MPs unfolding, leading MPs to establish a stable network-like structure [30].

Furthermore, the data of storage modulus (G′) and loss modulus (G′′) versus temperature was used to get information about gel processing and gel elasticity. The higher G' indicates a good protein entanglement. It can be seen from Fig. 4D that the G′ of untreated and UT-treated MPs emulsion slowly increased at temperature from 20 to 60 °C, then rapidly increased and reached a maximum value at 80 °C, and finally decreased at temperature from 80 to 95 °C. In addition, UT-treated MPs had higher G' than the control group. Similar results were reported by Pan et al. [21]. The UT altered the MPs’ crosslinking ability and enhanced the MPs gelation process [30]. Notably, G' of MPs reached the highest value after UT treatment for 15 min. These results indicate that UT had the potential in modifying MPs’ structure and enhancing rheological properties of MPs emulsion.

### 3.11 Gel properties

#### 3.11.1 LF-NMR analysis

The LF-NMR analysis was performed to get information about the water distribution in the gel network of MPs, and the results are displayed in Fig. 4 (E-G). The control group showed three different peaks, which indicate three different water forms including the bound (T_2b_), the immobilized (T_21_), and the free water (T_22_) (Fig. 4E).

It can be seen from Fig. 4F that T_2b_ was remarkably affected by the UT. MP gel had a lower T_2b_ after UT treatment for 10–30 min than the control group, demonstrating that its capacity to bind water was significantly improved. Notably, T_21_ and T_22_ of MP gel decreased after UT, indicating an improvement in the water-binding ability of protein after UT. Because of the thick and organized microstructure that formed in the UT-treated MP gel and the large amount of water that was entrapped, the amount of immobilized and free water loss in the MP gel network was reduced [40].

The bound, the immobilized, and the free water peak area fractions of MP gels were expressed as P_2b_, P_21_, and P_22_, respectively. The 15 min UT-treated samples had larger P_2b_ than other UT-treated samples (Fig. 4G), demonstrating a lower loss of bound water and a stronger ability to retain bound water. In addition, the lower content of P_21_ and higher content of P_22_ in the UT-treated samples indicate that the immobilized water changed to free water in the gel network. The cavitation action produced by UT promoted the MPs unfolding and the hydrophobic group's exposure, reducing the interaction between water and protein [40].

#### 3.11.2 WHC analysis

The WHC measurement could be used to study the ability of proteins to retain water. It can be seen from Fig. 4H that the WHC of MP gel was enhanced after UT treatment, demonstrating that UT could improve the ability of proteins to retain water. On the one hand, the residues of the exposed protein via hydrophobic interactions and hydrogen bonds increased the water-binding ability of protein [45]; on the other hand, the small particle size was beneficial for forming a fine gel network, which reduced water loss during centrifugation, leading to higher WHC [46].

#### 3.11.3 Morphlogical structure of gel

**Fig.5:**
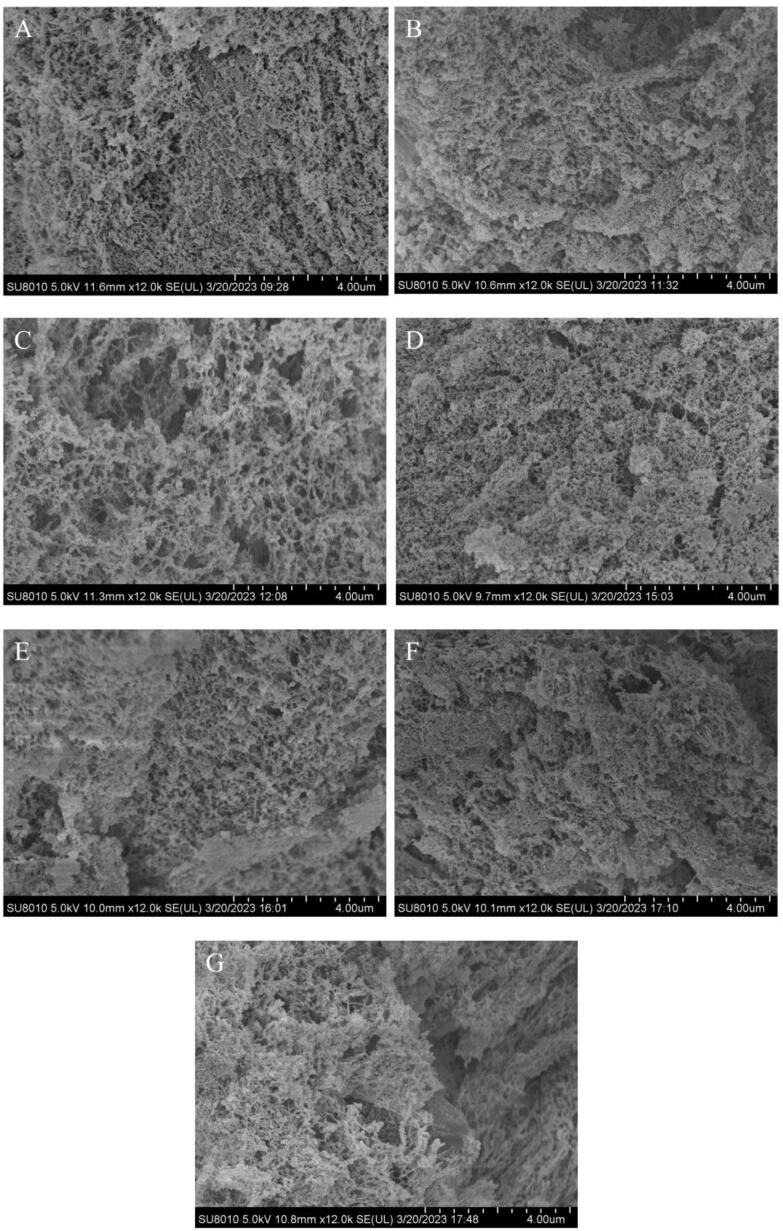
SEM (12000 × ) of MPs treated by UT. A, UT-0; B, UT-5; C, UT-10; D, UT-15; E, UT-20; F, UT-25; G, UT-30.

The SEM could be used to visualize the microstructure of gel for the untreated and UT-treated MP, and the images are displayed in Fig. 5. It can be seen from Fig. 5A that the three-dimensional MP gel had a coarse and discontinuous network with uneven pores. As the UT treatment prolonged from 0 to 10 min, the three-dimensional MP gel became more looser (Fig. 5B-C). A regular and ordered three-dimensional network structure was formed as the UT treatment extended to 15 and 20 min. This indicates that UT treatment enhanced the protein interaction and cross-linking, leading to a more ordered and compact three-dimensional network structure. Li et al. [42] found that exposure of hydrophobic groups on the protein surface could enhance the MPs molecules interactions via both disulfide bond and hydrophobic interactions, which formed a more compact three-dimensional network gels structure. However, when the UT duration was extended for more than 25 min, a disordered and inhomogeneous network gels structure developed, showing that the gel network was further damaged by the prolonged UT treatment. These results are consistent with findings of Zhang et al. [40] who found gel network was destroyed after excessive UT power. The long-time shock waves and turbulence produced highly reactive free radicals, causing a great degree of MP denaturation, which resulted in the formation of disordered aggregates, leading to the formation of a loose three-dimensional network structure [40].

### 3.12 Adsorption properties

**Fig. 6:**
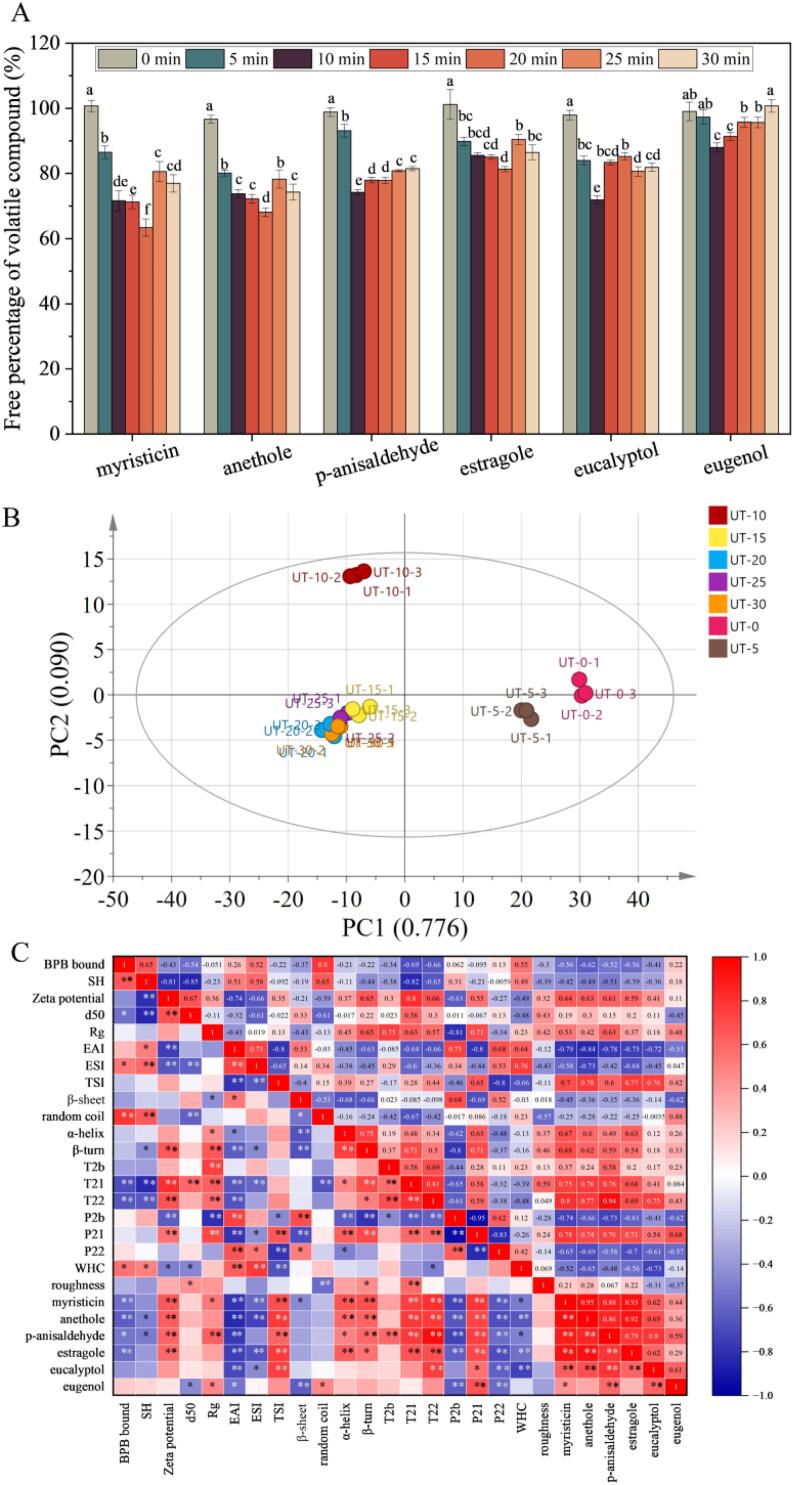
Adsorption characteristics of MPs treated by UT (A), principal component analysis (B) and correlation analysis (C) of myofibrillar protein structure, emulsifying properties, gel properties, and adsorption properties. Different small letters on columns indicate significant differences between different UT treatment durations (*P* < 0.05) for the same flavor compound. The correlation analysis was performed by Pearson's correlation analysis. The correlation coefficient is shown in different colors in the Figure, and the right legend is a color interval with different correlation coefficients. The darker colors indicate larger correlation coefficients (positive correlations are displayed in red and negative correlations in blue color). *, *P* < 0.05; **, *P* < 0.01. (For interpretation of the references to color in this figure legend, the reader is referred to the web version of this article.)

The free percentages of VCS in the headspace in presence of MPs were analyzed, and the results are displayed in Fig. 6A. The ability of protein to bind with flavor compounds was stronger and the free percentage was lower [2]. The free VCS percentages in the headspace of the untreated MPs were about 100%, demonstrating a lower ability of the MPs to bind with VCS. The ability of the MPs to bind with volatile compounds was affected by steric hindrance in MPs. It was reported that the MPs’ steric hindrance would prevent volatile compounds from entering hydrophobic regions of MPs, leading to a lower ability of the MPs to bind with VCS [47]. The alteration of MPs binding capability with VCS relied on the UT treatment duration. The characteristics of MPs, such as surface hydrophobicity, secondary structure, and surface morphology, as well as the characteristics of volatile substances, such as polarity, air/water partition coefficient, and steric hindrance effects, will work together to affect the ability of proteins to bind volatile substances [7].

The UT treatment for 0 to 30 min could improve the ability of the MPs to bind with volatile compounds such as myristicin, anethole, p-anisaldehyde, estragole, and eucalyptol. Because more active sites were exposed as a result of the protein unfolding brought on by UT cavitation, more volatiles interacted with proteins. Interestingly, no significant difference in the ability of eugenol to combine with proteins after UT treatment for 0, 25, and 30 min. This may be due to that surface tension and protein–protein interactions changed the partitioning of eugenol in the vapor phase, leading to lower protein-eugenol interactions [7].

Notably, differences in the ability of the MPs to bind with VCS were found during UT treatment. The percentages of p-anisaldehyde, eucalyptol, and eugenol reached their lowest after UT treatment for 10 min. In addition, the percentages of myristicin, anethole, and estragole reached their lowest after UT treatment of MPs for 20 min. This phenomenon may be attributed to the differences in the VCS’ structure led to the difference in the ability of proteins to retain VCS [47]. These results indicated that the protein maintained a higher VCS content after UT treatment, which is beneficial to improve flavor control in meat products [2].

### 3.13 PCA and correlation analysis

The PCA analysis was performed to verify the differences among the samples, and the results are shown in Fig. 6B. Two principal components, namely PC1 (77.6%), and PC2 (9.0 %) were extracted, which showed the reliability of sample information. The untreated and UT-treated samples were in different quadrants, indicating that UT could change the properties of MP samples. In addition, the 5 and 10 min UT-treated samples were in the upper right and left quadrants, respectively, indicating significant differences among properties of both samples. However, the 20, 25, and 30 min UT-treated samples were grouped in the lower left quadrant, indicating insignificant differences among their properties.

To explore the relationship among structure, rheological, emulsifying, and gel properties of MPs, as well as the ability of the MPs to bind VCS, correlation analysis was performed. It can be seen from Fig. 6C that the ability of eugenol to combine with proteins was highly correlated with β-sheet, P_21_, P_22_, and P_2b_, and correlated with d50, Rg, EAI, and random coil. The EAI, TSI, T_22_, and P_22_ were highly correlated with the ability of myristicin, anethole, p-anisaldehyde, estragole, and eucalyptol to combine with proteins. In addition, the ability of myristicin, anethole, and estragole to bind with proteins was highly correlated with surface hydrophobicity, ζ-potential, and α-helix. The UT treatment changed the conformation, emulsifying, and gel properties of MPs, which affect the ability of VCS to bind with MPs.

Meanwhile, ζ-potential was found to have a negative correlation with SH, EAI, and ESI, and a positive correlation with d_50_. The depolymerization of agglomerates and masses into smaller particles, and the exposure of sulfhydryl and negatively charged groups on the surface of MPs, may improve the emulsion stability. The ability of myristicin, anethole, p-anisaldehyde, and estragole to bind with MPs was found to have a negative correlation with TSI, and a positive correlation with EAI. The UT treatment led to MPs unfolding and changed the surface-to-volume ratio, which enhanced the dispersion stability and exposure of binding sites, resulting in a high physically stable protein-VCS combination. The obtained results demonstrated that the increase in EAI and decrease in TSI could enhance the binding ability of MPs with VCS.

## 4 Conclusion

UT enhanced surface hydrophobicity, SH content, and absolute ζ-potential value of chicken breast meat MPs. Microscopy analysis showed the formation of MPs aggregates with small particle size following UT treatment. The emulsifying properties and physical stability of MPs’ emulsion improved after UT treatment. Additionally, UT treatment resulted in enhanced MPs gel network structure and stability. Also, the ability of MPs to bind to flavor substances from spices was enhanced depending on duration of UT treatment because of changes in the structural, physicochemical, and functional properties of MPs. The ability of myristicin, anethole, and estragole to bind to MPs was highly correlated with surface hydrophobicity, ζ-potential value, and α-helix content of MPs. The findings of this study could be used to better understand how the characteristics of MPs vary as meat products are processed and how this affects their capacity to bind to VCS, enhancing flavor retention in processed meat products. However, more studies are required to understand the mechanisms underlying the binding of meat and flavors under various practical processing conditions.

## CRediT authorship contribution statement

**Xiangxiang Sun:** Investigation, Validation, Conceptualization, Writing – review & editing. **Yumei Yu:** Methodology, Software. **Ahmed S.M. Saleh:** Data curation, Investigation, Writing – review & editing. **Xinyu Yang:** Data curation, Formal analysis. **Jiale Ma:** Investigation, Methodology. **Ziwu Gao:** Methodology. **Wenhao Li:** Formal analysis, Methodology. **Zhenyu Wang:** Conceptualization, Funding acquisition, Project administration, Supervision. **Dequan Zhang:** Methodology, Writing – review & editing, Supervision.

## Declaration of Competing Interest

The authors declare that they have no known competing financial interests or personal relationships that could have appeared to influence the work reported in this paper.
